# Circular RNA circKIF4A Sponges miR-375/1231 to Promote Bladder Cancer Progression by Upregulating NOTCH2 Expression

**DOI:** 10.3389/fphar.2020.00605

**Published:** 2020-05-08

**Authors:** Ying-rui Shi, Zheng Wu, Kun Xiong, Qian-jin Liao, Xu Ye, Pei Yang, Xiong-bing Zu

**Affiliations:** ^1^ Department of Urology, Xiangya Hospital, Central South University, Changsha, China; ^2^ Department of Radiation Oncology, Hunan Cancer Hospital & the Affiliated Cancer Hospital of Xiangya School of Medicine, Central South University, Changsha, China; ^3^ Central Laboratory, Hunan Cancer Hospital & the Affiliated Cancer Hospital of Xiangya School of Medicine, Central South University, Changsha, China

**Keywords:** circKIF4A, circular RNAs, NOTCH2, miR-375, bladder cancer

## Abstract

Circular RNAs (circRNAs) have been found to be important mediators of many biological processes in the growth and metastasis of various cancers. However, the potential roles of most circRNAs in the progression of bladder cancer remain unclear. In this research, we investigate the role of circKIF4A (hsa_circ_0007255) in the development and progression of bladder cancer. Detected by qRT-PCR analysis, circKIF4A was significantly upregulated in bladder cancer tissues and cell lines. We conducted CCK-8, colony-formation, transwell and mouse xenograft assays to explore the function of circKIF4A in bladder cancer. Functionally, knockdown of circKIF4A inhibited the proliferation and colony-formation ability of bladder cancer cells. Migration and metastatic ability were dramatically decreased after transfection with small interfering RNA targeting circKIF4A in both *in vitro* and *in vivo* assays. Mechanically, luciferase reporter assays and RNA immunoprecipitation assays were carried out to elucidate the underlying molecular mechanism of circKIF4A. The results revealed that circKIF4A sponges miR-375/1231 to promote bladder cancer progression by upregulating NOTCH2. Generally, our research unveils the essential role of circKIF4A-miR-375/1231-NOTCH2 axis in bladder cancer progression possibly *via* the competing endogenous RNA mechanism.

## Introduction

Bladder cancer is the most prevalent malignancy of the urinary system and one of the most frequently diagnosed cancers worldwide (Antoni et al., 2017). According to cancer statistics in China, the mortality and morbidity rates of bladder cancer rank first among all malignancies of the urinary system (Chen et al., 2016). Despite progress and advances in the early diagnosis and systematic treatment of bladder cancer, some patients still suffer from recurrence or metastatic bladder cancer diseases (Dreicer, 2017). According to the depth of tumor invasion, bladder cancer can be divided into two different subtypes: non-muscle invasive tumor and muscle-invasive tumor (van Rhijn et al., 2009). Accounting for approximately 20–30% of all bladder tumors, patients with muscle-invasive tumors have a higher risk of metastasis and a worse prognosis (Dy et al., 2017). Therefore, it is of great importance to understand the underlying mechanism and discover novel therapeutic targets.

Circular RNAs (circRNAs) are a novel class of endogenous noncoding RNAs with a circular structure (Jeck et al., 2013). circRNAs are derived from the back-splice of exons or introns of messenger RNAs and are stable in cells. Compared to linear RNAs, circRNAs are more abundant and have no cap or tail in mammalian tissues (Jeck and Sharpless, 2014). Once regarded as the byproduct of incorrect transcription of genes, circRNAs are now recognized as important regulators of multiple biological intracellular processes, including the development and progression of cancers (Bach et al., 2019).

Different RNAs (mRNA, pseudogene, lncRNA, etc.) can act as competitive endogenous RNAs (ceRNAs) by competing for microRNA (miRNA) binding in the cytoplasm (Tay et al., 2014). As one type of novel noncoding RNA, circRNAs have been identified as ceRNAs that act as miRNA sponges, leading to the loss of miRNA functions (Wang et al., 2016). For example, the most well-known and well-studied circRNA ciRS-7 promotes the proliferation and metastasis of different tumors by sponging miR-7 (Memczak et al., 2013; Weng et al., 2017; Su et al., 2019; Zhao et al., 2019; Zou et al., 2019a). circFBXW7 is downregulated in tumor tissues and suppresses cancer cell growth and metastasis by encoding the 21 kDa novel protein FBXW7-185aa and sponging miR-197-3p in glioma and breast cancer (Yang et al., 2018; Ye et al., 2019). Circular RNA PRMT5 sponges miR-30c and promotes bladder cancer progression by inducing epithelial-mesenchymal transition (EMT) (Chen et al., 2018). CircKIF4A and circRAD18 were identified as pro-cancerous factors in triple-negative breast cancer though the mechanism of competing endogenous RNAs (Tang et al., 2019; Zou et al., 2019b). However, the potential functions and the underlying molecular mechanism of most circRNAs remain unknown in bladder cancer.

In this study, we investigated the biological role and functions of circKIF4A in bladder cancer. CircKIF4A was upregulated in bladder cancer tissues and cells. Silencing of circKIF4A significantly suppressed the growth and metastatic ability in bladder cancer cells and animals. We performed luciferase reporter assays and RNA immunoprecipitation assays to demonstrate the molecular mechanism of circKIF4A in bladder cancer. Overall, this study discovered the function of the circKIF4A-miR-375/1231-NOTCH2 axis in bladder cancer progression.

## Methods

### Patient Samples and Ethical Standards

Fresh primary bladder cancer tissues and adjacent tissues were collected at Xiangya Hospital and frozen immediately after resection. This study was approved by the Ethics Committee of Xiangya Hospital and performed in accordance with the Declaration of Helsinki. Written informed consent was obtained from all patients. Animal experiments were approved and performed according to the guidelines of the Institutional Animal Care and Use Committee of Xiangya Hospital.

### Cell Culture

All cell lines used in this study, namely, HEK293T, 5637, RT-112 and BIU-87, were purchased from the American Type Culture Collection (ATCC, USA). Cells were cultured according to the instructions of the manufacturer and passaged for less than 6 months. Cell authenticity was verified by DNA fingerprinting.

### Quantitative Real-Time PCR (qRT-PCR) and Transfection

TRIzol (Invitrogen) was used to extract total RNA. Isolation of the nuclear and cytoplasmic fractions of cellular RNA was performed with NE-PER Nuclear and Cytoplasmic Extraction Reagents (Thermo Scientific). SYBR Premix Ex Taq (Takara) was used to perform qRT-PCR. Transfection of cell lines was conducted with Lipofectamine 3000 (Invitrogen). The primers were as follows: 5′-GAGGTACCCTGCCTGGATCT-3′ (forward), 5′-TGGAATCTCTGTAGGGCACA-3′ (reverse) for circKIF4A; 5′-TTAATTCCGATAACGAACGAGA-3′ (forward), 5′-CGCTGAGCCAGTCAGTGTAG-3′ (reverse) for 18S; 5′-AGCGAGCATCCCCCA AAGTT-3′ (forward), 5′-GGGCACGAAGGCTCATCATT-3′ (reverse) for β-actin; 5′-GGAGCGAGATCCCTCCAAAAT-3′ (forward), 5′-GGCTGTTGTCATACTTCTCAT GG-3′ (reverse) for GAPDH; and 5′-TCCAGCAATCGAACCCCAG-3′ (forward), 5′-CTTGTCTGCGAATCCGTAATGAT-3′ (reverse) for NOTCH2.

### Colony Formation Assay

A total of 1 × 10^3^ cells were plated and incubated in each well of a 6-well plate. After incubation at 37°C for 10 days, colonies were fixed with methanol and stained with 0.3% crystal violet.

### Cell Counting Kit-8 (CCK-8) Assay

A total of 5 × 10^3^ cells were seeded into a 96-well plate. Ten milliliters of CCK-8 solution (Dojindo Laboratories, Japan) was added to each well on day four. After incubation at 37°C for 1 h, the absorbance at a wavelength of 450 nM was measured.

### Transwell Assay

Migration chambers (BD Biosciences) were used to conduct transwell assays. In total, 10^4^ cells were added to the upper chambers (medium without serum), and medium (containing serum) was added to the lower chambers.

### Luciferase Reporter Assay

RT-112 and BIU-87 bladder cancer cells were seeded into 96-well plates with 3 × 10^3^ cells per well. After the transfected with constructed plasmids (circKIF4A-wt/mut or NOTCH2 3′-UTR-wt/mut) and miRNA mimics or inhibitors for 48 h, relative luciferase activity was evaluated by the dual-luciferase reporter assay system kit (Promega). All the procedures were conducted according to the manufacturer’s instructions.

### RNA Immunoprecipitation

RT-112 and BIU-87 bladder cancer cells were cotransfected with MS2bs-Rluc, MS2bs-circKIF4A and MS2bs-circKIF4Amt. RNA immunoprecipitation was conducted with a Magna RIP RNA-Binding Protein Immunoprecipitation Kit (Millipore). RNA immunoprecipitation assays for Ago2 were conducted with an anti-Ago2 antibody (Millipore). The levels of circKIF4A, miR-375/1231 and NOTCH2 were determined after purification.

### Western Blot Analysis

Total cell protein was extracted by RIPA lysis buffer. The primary antibodies anti-NOTCH1 (1:1,000, CST, USA), anti-NOTCH2 (1:1,000, CST, USA), anti-PI3K (1:1,000, Affinity, USA), anti-AKT (1:1,000, CST, USA), anti-phospho-AKT (1:1,000, CST, USA), anti-KIF4A (1:1,000, Abcam, USA) and anti-β-actin (1:1,000, Affinity, USA) were used to detect specific proteins.

### Mouse Xenograft Model

A total of 2 × 10^7^ RT-112 and BIU-87 bladder cancer cells were subcutaneously injected into the dorsal flanks of BALB/c nude mice (4 weeks old, female). The mice were randomly grouped (five mice/group) and treated with an intratumoral injection (40 μl si-NC or si-circKIF4A) every 4 days after tumors become palpable. After 4 weeks, the mice were euthanized, and the tumors were weighed and recorded.

For the lung metastasis assays, 2 × 10^5^ cells (transfected with si-NC or si-circKIF4A) were injected through the tail veins of mice (five mice/group). The lungs of mice were excised 8 weeks later, and the number of metastatic nodules was counted under microscopy after hematoxylin and eosin (HE) staining.

### Statistical Analysis

All statistical analyses in this study were performed using SPSS 24.0 software (SPSS Inc., Chicago, IL, USA). Quantitative data are presented as the mean ± standard deviation (SD). Groups were compared using a t-test. *P <*0.05 was considered statistically significant.

## Results

### circKIF4A Promotes Bladder Cancer Growth and Metastasis *In Vitro*

**Figure 1:**
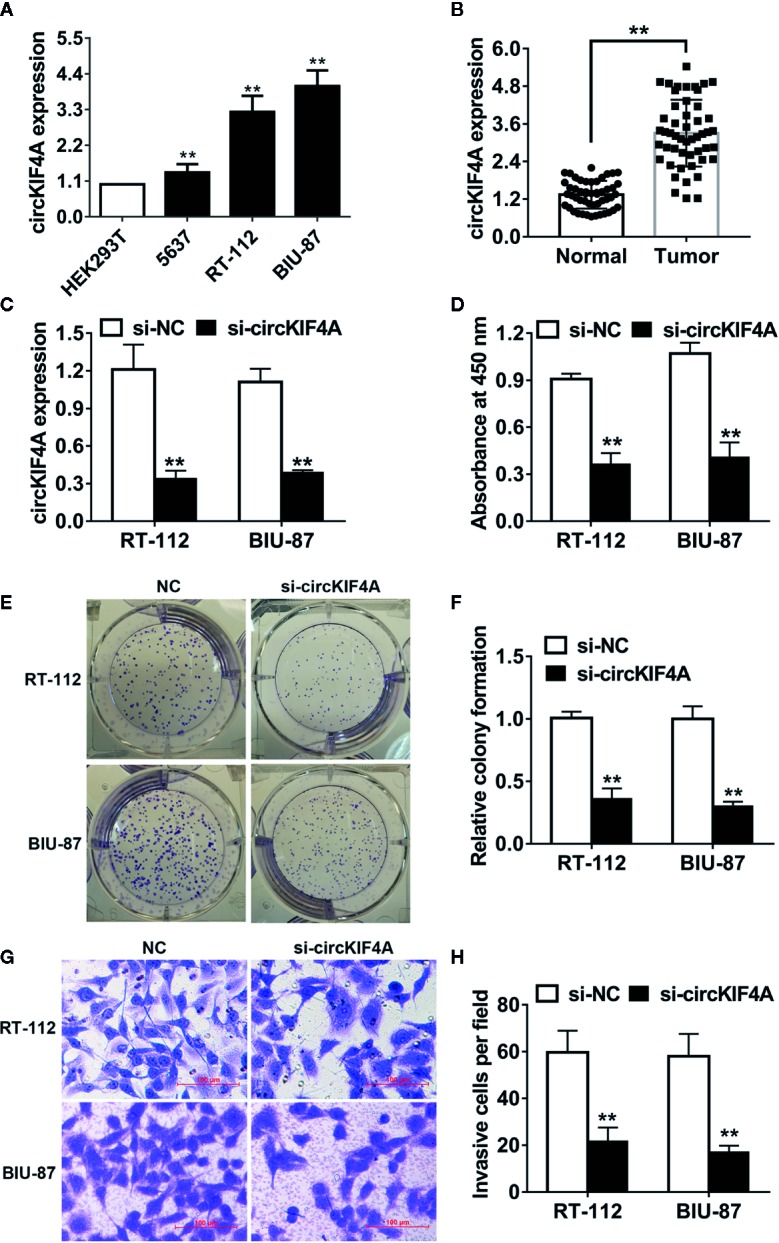
circKIF4A promotes bladder cancer growth and metastasis in vitro. **(A)** The relative expression level of circKIF4A in bladder cancer cell lines. **(B)** The expression level of circKIF4A was validated in 50 paired bladder cancer and adjacent normal tissues. **(C)** The knockdown of circKIF4A was assessed by qRT-PCR. **(D)** CCK-8 assay was conducted to detect cell proliferation. **(E, F)** Colony formation assays were conducted. **(G, H)** Inhibition of circKIF4A reduced the invasion ability in the transwell assay. ^**^P < 0.01.

CircKIF4A (hsa_circ_0007255) was overexpressed in 5637, RT-112 and BIU-87 bladder cancer cell lines compared to HEK293T cells (**Figure 1A**). We next validated the expression level of circKIF4A in 50 paired bladder tumor tissues and adjacent normal tissues. We found that circKIF4A was upregulated in bladder cancer tissues (**Figure 1B**). To explore the function of circKIF4A in bladder cancer, we designed a small interfering RNA to knock down circKIF4A and validated its efficiency in two cell lines (**Figure 1C**). Silencing circKIF4A suppressed the proliferation of RT-112 and BIU-87 bladder cancer cells, as demonstrated in CCK-8 assays (**Figure 1D**). Knockdown of circKIF4A significantly inhibited the colony formation ability of bladder cancer cells (**Figures 1E, F**). In transwell assays, inhibition of circKIF4A reduced the invasion ability of bladder cancer cells (**Figures 1G, H**).

### Downregulation of circKIF4A Suppresses the Proliferation of Bladder Cancer *In Vivo*

**Figure 2:**
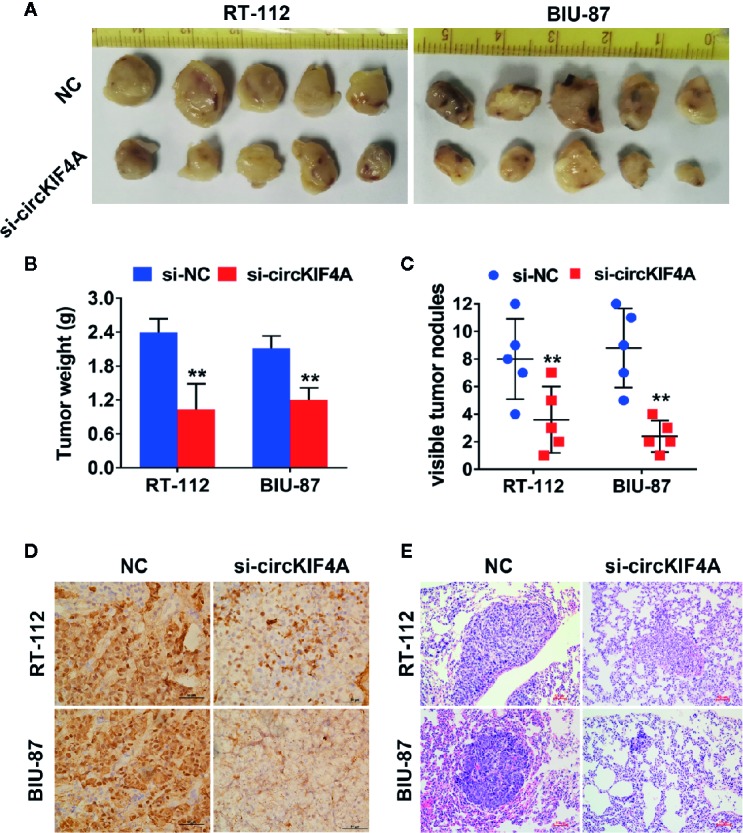
Downregulation of circKIF4A suppresses the proliferation and metastasis of bladder cancer in vivo. **(A)** A mouse xenograft model was established using RT-112 and BIU-87 cell lines. **(B)** Tumor volumes were weighed. **(C)** The number of metastatic sites in the lung was counted and recorded. **(D)** Xenograft tumors were stained with Ki-67 antibody for immunohistochemistry analysis, and representative images are presented. **(E)** HE-stained sections of lung metastases were imaged under a microscope. ^**^P < 0.01.

To further evaluate the functions of circKIF4A *in vivo*, mouse xenograft models were established. The results revealed that silencing circKIF4A could reduce the tumor volume established by RT-112 and BIU-87 bladder cancer cell lines (**Figures 2A, B**). Additionally, the expression of the proliferation biomarker Ki67 was significantly reduced in tumor tissues in the si-circKIF4A group (**Figure 2D**). Next, lung metastasis assays were conducted to evaluate the influence of circKIF4A on metastatic ability. Inhibition of circKIF4A significantly reduced the number of lung metastases (**Figures 2C, E**).

### circKIF4A Functions as a Sponge for miR-375 and miR-1231

**Figure 3:**
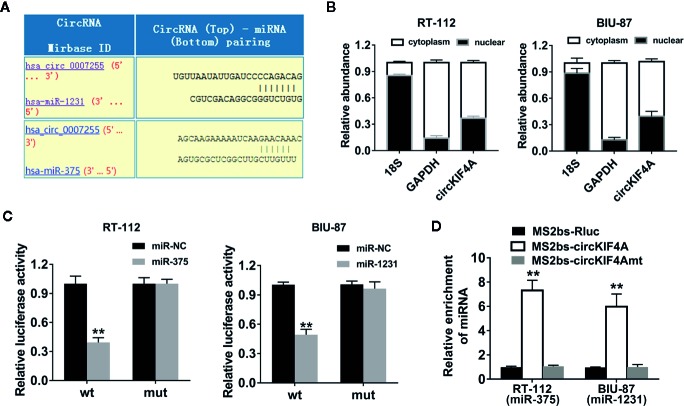
circKIF4A functions as a sponge for miR-375 and miR-1231. **(A)** Prediction of the binding sites of miR-375 and miR-1231 in the circKIF4A sequence (https://circinteractome.nia.nih.gov). **(B)** The expression levels of 18S, GAPDH and circKIF4A in nuclear and cytoplasmic fractions. **(C)** Luciferase reporter assays were conducted. RT-112 and BIU-87 cells were cotransfected with miR-375/1231 mimics and circKIF4A wild-type or mutant luciferase reporter. **(D)** MS2-based RIP assay in RT-112 and BIU-87 cells transfected with MS2bs-circKIF4A, MS2bs-circKIF4A-mt or MS2bs-Rluc. ^**^P < 0.01.

We used the Circular RNA Interactome website tool to predict potential circRNA and miRNA interactions (https://circinteractome.nia.nih.gov). Among the candidates, miR-375 and miR-1231 were predicted to have the potential to interact with circKIF4A (**Figure 3A**). miR-375 and miR-1231 were both found to be tumor suppressors in multiple types of cancers (Zheng et al., 2016; Selth et al., 2017; Zhao et al., 2018; Shang et al., 2019; Xu et al., 2019). CircKIF4A was mostly localized in the cytoplasm, revealing that it could interact with multiple miRNAs, which are predominantly located in the cytoplasm (**Figure 3B**). The relative luciferase activity was decreased after cotransfection with the wild-type reporter and miR-375 or miR-1231 mimics as evaluated by dual luciferase reporter assays (**Figure 3C**). To further explore the interactions between circKIF4A, miR-375 and miR-1231, RNA immunoprecipitation (RIP) assays were conducted. The results revealed that miR-375 and miR-1231 were enriched in the MS2bs-circKIF4A group (**Figure 3D**).

### circKIF4A Promotes Bladder Cancer Progression *via* the circKIF4A-miR-375/1231-NOTCH2 Axis

**Figure 4:**
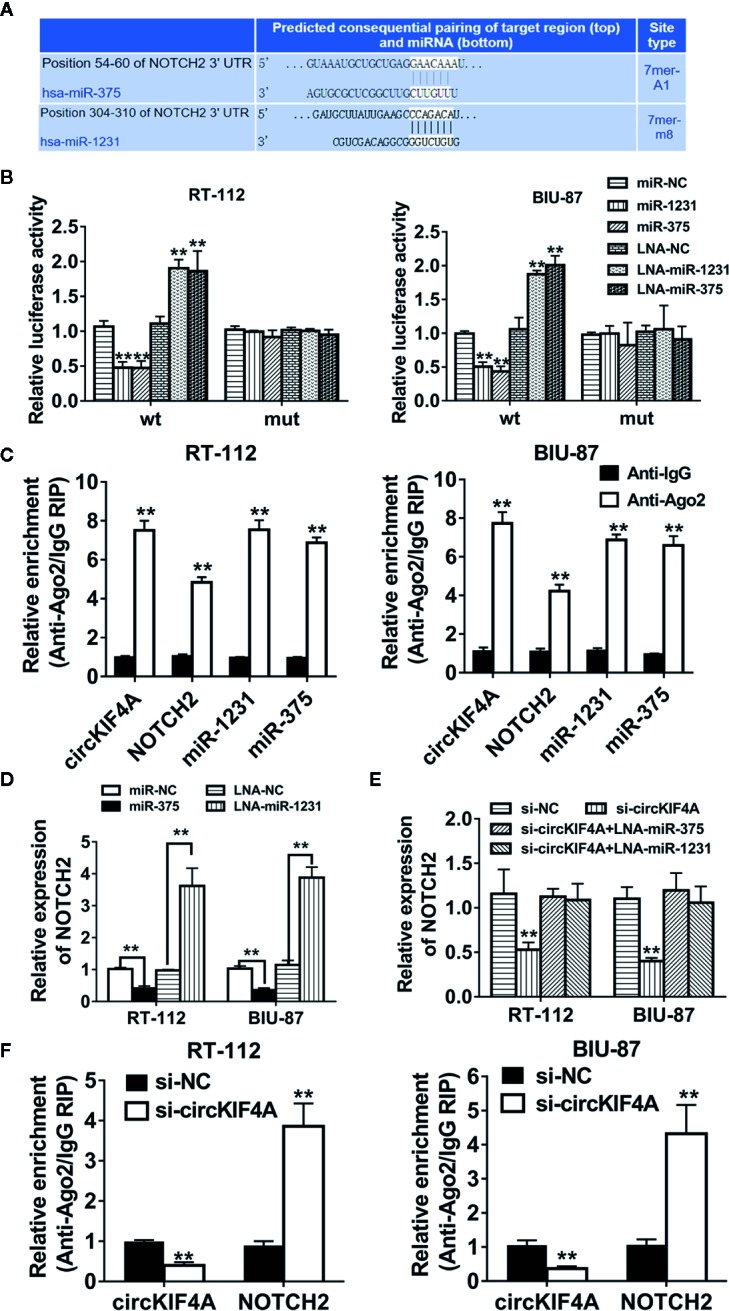
NOTCH2 is the co-target of miR-375 and miR-1231. **(A)** Predicted binding sites of miR-375 and miR-1231 in the 3′-UTR of NOTCH2 according to the TargetScan algorithm (http://www.targetscan.org). **(B)** Luciferase reporter assays were conducted. RT-112 and BIU-87 cells were cotransfected with miR-375/1231 mimics, locked nucleic acid (LNA) and circKIF4A wild type or mutant luciferase reporter. **(C)** Enrichment of circKIF4A, NOTCH2, miR-375 and miR-1231 with Ago2 assessed by RIP assay. **(D)** The expression level of NOTCH2 was decreased after transfection with miR-375/1231 mimics. The expression of NOTCH2 was increased after knockdown of miR-375/1231. **(E)** Influence of circKIF4A on the expression of NOTCH2 detected by qRT-PCR analysis. **(F)** Enrichment of Ago2 for circKIF4A was decreased, while NOTCH2 was increased after knockdown of circKIF4A. **P < 0.01.

**Figure 5:**
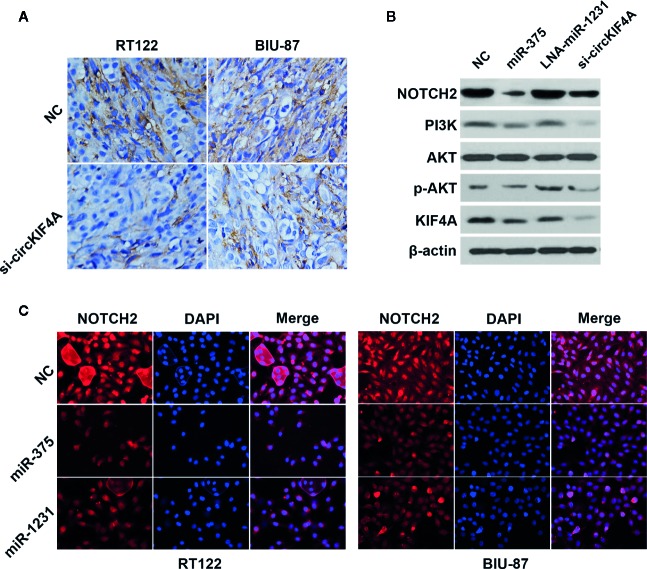
circKIF4A promotes bladder cancer progression *via* the circKIF4A-miR-375/1231-NOTCH2 axis. **(A)** Representative immunohistochemistry images of NOTCH2 expression in a mouse xenograft model. **(B)** Western blot analysis was conducted to evaluate the influence of miR-375, miR-1231 and circKIF4A on NOTCH2, the PI3K-AKT signaling pathway and KIF4A in the RT-112 cell line. **(C)** Immunofluorescence staining of NOTCH2 after transfection with miR-375 or miR-1231 mimics in RT-112 and BIU-87 cells.

Next, we used the TargetScan algorithm (http://www.targetscan.org) to predict the co-target of miR-375 and miR-1231, and NOTCH2 was identified as the candidate target oncogene (**Figure 4A**). NOTCH2 has been found to be a robust oncogene in bladder cancer by promoting cell proliferation and metastasis through epithelial-to-mesenchymal transition, cell cycle progression, and maintenance of stemness (Maraver et al., 2015; Hayashi et al., 2016; Goriki et al., 2018). We conducted luciferase reporter assays and RNA immunoprecipitation assays to confirm the interaction between the 3′-UTR of NOTCH2 mRNA, miR-375 and miR-1231. The luciferase reporter assay revealed that the relative luciferase activity was reduced after cotransfection with miR-375/1231 mimics and the wild-type 3′-UTR-NOTCH2 reporter (**Figure 4B**). In addition, Ago2-related RIP assays revealed that circKIF4A, NOTCH2 and miR-375/1231 were enriched for Ago2 in RT-112 and BIU-87 bladder cancer cells (**Figure 4C**). Overexpression of miR-375 or miR-1231 decreased the expression level of NOTCH2, and inhibition of miR-375 or miR-1231 increased NOTCH2 expression (**Figure 4D**). Silencing circKIF4A significantly reduced NOTCH2 expression, while this effect could be reversed by blocking miR-375/1231 (**Figure 4E**). Downregulation of circKIF4A remarkably increased NOTCH2 enrichment for Ago2 (**Figure 4F**). We assessed the expression of NOTCH2 in mouse tumor xenografts by immunohistochemical staining and found that NOTCH2 expression in the si-circKIF4A group was significantly decreased (**Figure 5A**). Western blot analysis showed that inhibition of circKIF4A decreased the expression of NOTCH2 and inhibited the PI3K-AKT signaling pathway in the RT-112 cell line (**Figure 5B**). Immunofluorescence staining revealed that overexpression of miR-375 and miR-1231 could decrease the expression of NOTCH2 in RT-112 and BIU-87 cells (**Figure 5C**).

## Discussion

CircRNAs are a novel type of noncoding RNA that has become one of the hottest topics in biomedicine. Currently, high-throughput sequencing technology and bioinformatics algorithms are frequently used by researchers to identify and characterize thousands of different circRNAs (Shen et al., 2019). Scientists have realized that circRNAs are not simply junk byproducts of pre-mRNA splicing but are important regulators of biological processes (Chen, 2016). In recent years, an increasing number of circRNAs have been identified and studied in different kinds of diseases, especially in cancers (Geng et al., 2019). Bladder cancer is the most pervasive malignancy of the urinary system and one of the most frequently diagnosed cancers worldwide (Antoni et al., 2017). CircRNAs have been proven to have important functions in the development and progression of bladder cancer. Circular RNA PRMT5 promotes the metastasis of bladder cancer by inducing EMT and can also be secreted into exosomes and detected in urine (Chen et al., 2018). CircUBXN7 and circ5912 were confirmed as tumor suppressors of bladder cancer by sponging miR-1247 and inducing MET, respectively (Liu et al., 2018; Su et al., 2019). However, the potential role of most circRNAs in the progression of bladder cancer remains unclear.

In this study, we explored the biological role and functions of circKIF4A in bladder cancer. CircKIF4A was upregulated in bladder cancer tissues compared to normal bladder tissues. Knockdown of circKIF4A significantly suppressed the growth and metastatic ability in bladder cancer cells and animals. miR-375 and miR-1231 were both predicted to interact with circKIF4A. We performed luciferase reporter assays and RNA immunoprecipitation assays to demonstrate the molecular mechanism of circKIF4A in bladder cancer. The results showed that circKIF4A could sponge miR-375 and miR-1231 to relieve the inhibition of NOTCH2.

According to previous studies, miR-375 and miR-1231 have been confirmed as tumor suppressors in many types of cancer. By targeting YAP1, miR-375 was found to inhibit prostate cancer growth, which was shown to be directly repressed by the EMT transcription factor ZEB1 (Selth et al., 2017). The ASH1-miR-375-YWHAZ signaling axis was found to suppress tumor progression in hepatocellular carcinoma (Zhao et al., 2018). miR-375 reverses chemoresistance by targeting YAP1 and SP1 in colorectal cancer (Xu et al., 2019). miR-1231 induces PTPN11 degradation and promotes pancreatic cancer growth (Zheng et al., 2016). Moreover, exosomal miRNA-1231 can be secreted by bone marrow mesenchymal stem cells, which can inhibit the growth of pancreatic cancer cells (Shang et al., 2019). Additionally, NOTCH2 was proven to be a robust oncogene in bladder cancer by activating the Notch signaling pathway (Hayashi et al., 2016). In our study, NOTCH2 was confirmed to be the co-target of miR-375 and miR-1231 in bladder cancer. circKIF4A could increase the expression of NOTCH2 though the competing endogenous RNA mechanism.

In conclusion, the present study investigated the biological function of circKIF4A in bladder cancer. Our study revealed the pivotal role of the circKIF4A-miR-375/1231-NOTCH2 axis in bladder cancer progression possibly *via* the competing endogenous RNA mechanism. Thus, circKIF4A could be a novel therapeutic target for bladder cancer treatment.

## Data Availability Statement

The raw data supporting the conclusions of this article will be made available by the authors, without undue reservation, to any qualified researcher.

## Ethics Statement

This study was approved by the Ethics Committee of Xiangya Hospital and performed in accordance with the Declaration of Helsinki. Written informed consent was obtained from all patients. Animal experiments were approved and performed according to the guidelines of the Institutional Animal Care and Use Committee of Xiangya Hospital.

## Author Contributions

X-BZ and Y-RS designed the experiments. Y-RS, ZW and KX performed the experiments. Q-JL, XY and PY analyzed and interpreted the data. Y-RS was the major contributors in writing the manuscript. X-BZ reviewed and revised the paper. All authors read and approved the final manuscript.

## Funding

Our study was funded by the National Natural Science Foundation of China (No. 81572523) and the National Key Research and Development Program of China (2016YFC0902603).

## Conflict of Interest

The authors declare that the research was conducted in the absence of any commercial or financial relationships that could be construed as a potential conflict of interest.

The reviewer J-YY declared a shared affiliation, with no collaboration, with the authors to the handling editor.

## Abbreviations

circRNA: circular RNA; ceRNA: competing endogenous RNAs; KIF4A, kinesin family member 4A; miRNAs: microRNAs; NOTCH2, notch receptor 2; SD: standard deviation; siRNA: small interfering RNA; RIP: RNA immunoprecipitation.
